# The Contribution of Multiplex Polymerase Chain Reaction in the Diagnosis of Central Nervous System Infections in Intensive Care Units

**DOI:** 10.7759/cureus.35338

**Published:** 2023-02-22

**Authors:** Fayrouz Debbagh, Sara Harrar, Fatima Babokh, Asma Lamrani Hanchi, Nabila Soraa

**Affiliations:** 1 Microbiology Laboratory, Faculty of Medicine and Pharmacy, Mohammed VI University Hospital of Marrakech, Cadi Ayyad University, Marrakech, MAR; 2 Biochemistry-Toxicology Laboratory, Avicenna Military Hospital, Marrakech, MAR; 3 Microbiology Department, Arrazi Hospital, Mohammed VI University Hospital of Marrakech, Marrakech, MAR; 4 Biology Department/Parasitology and Mycology Laboratory, Faculty of Medicine and Pharmacy, Mohammed VI University Hospital of Marrakech, Cadi Ayyad University, Marrakech, MAR; 5 Microbiology Laboratory, Arrazi Hospital, Mohammed VI University Hospital of Marrakech, Marrakech, MAR

**Keywords:** filmarray, impact, children, adults, rapid diagnosis, intensive care units, biofire, multiplex pcr, central nervous system infections

## Abstract

Introduction

The aim of this study was to evaluate the contribution and limits of BioFire® FilmArray® meningitis/encephalitis panel (FA MEP) polymerase chain reaction (PCR) (bioMérieux, Marcy-l'Étoile, France) (product references: LLC RFIT-ASY-0118) coupled with bacterial and fungal culture in the diagnosis of central nervous system infections (CNSIs).

Methods

This was a retrospective observational study including all patients (adults and children) hospitalized in the intensive care units (ICUs) of a Moroccan university hospital, who benefited from multiplex PCR on a cerebrospinal fluid (CSF) sample.

Results

A total of 112 PCRs were performed, with a positivity rate of 18%. Bacterial etiology was the most frequent (70%), represented mainly by *Streptococcus pneumoniae* (45%), followed by viruses (25%), with four isolates of *Herpes simplex virus (HSV) 1*. On 94 samples, there was an agreement between the culture and PCR results. Their discordance was found in 18 cases, including 16 suspected CNSIs recovered only by PCR and two diagnoses confirmed only by bacterial culture.

Conclusion

This study revealed the significant impact of multiplex PCR on the early and targeted diagnostic and therapeutic management of infectious meningitis and meningoencephalitis in intensive care unit patients.

## Introduction

Central nervous system infections (CNSIs) are serious conditions that continue to have important clinical consequences. They are associated with a high morbidity and mortality rate, particularly meningitis, which can be life-threatening in the absence of prompt administration of adequate antimicrobial therapy [1,2]. This mortality rate ranges from 5% to 30% for meningitis and from 9% to 12% for meningoencephalitis [3]. At the same time, significant sequelae can occur in 30%-50% of cases, such as motor deficits of the limbs, hearing disorders, convulsions, and cognitive deficits [1]. These infections also have a high cost of care, both short-term and long-term [4].

The clinical manifestations of these diseases are diverse and nonspecific, making etiological diagnosis difficult [5,6]. The first cytological and biochemical parameters of the cerebrospinal fluid (CSF) study may point to bacterial or other origins, but without being specific [7]. However, the etiology is not always identified and would be related to the lack of targeted tests, the large number of possible infectious causes, and the fact that 10% of suspected cases are related to noninfectious causes [8]. Also, the fact that postinfectious encephalitis has specific symptoms explains why the cause of half of infectious encephalitis is still unknown [9].

The standard techniques for the etiological diagnosis of these neuromeningeal diseases may present some insufficiencies. Indeed, the culture performed if bacterial or fungal meningitis is suspected could either delay the management of these at-risk patients, with a delay of results between two and five days, or give false-negative results when dealing with fastidious bacteria or in the presence of probabilistic antibiotic therapy, causing decapitated infection. For viruses, the real-time molecular biology technique only allows the targeted search for a given viral agent, requiring precise clinical orientation to guide the diagnostic approach [8].

In view of the important clinical consequences linked to the delay in diagnosis, new molecular tools responding to the diagnosis by syndromic approach have been developed, having as a principle the simultaneous detection of the bacterial, viral, or fungal genome by a multiplex polymerase chain reaction (PCR) technique. These tools have made it possible to obtain fast, precise results and solve some problems that came up with traditional methods.

In this context, the objective of this study was to define the place of the multiplex BioFire® FilmArray® meningitis/encephalitis panel (FA MEP) polymerase chain reaction (PCR) (bioMérieux, Marcy-l'Étoile, France) (product references: LLC RFIT-ASY-0118) among the prescriptions of microbiological examinations at the level of different intensive care units (ICUs) at the Mohammed VI University Hospital of Marrakech between 2018 and 2020 and evaluate its contribution, coupled with bacterial and fungal cultures, in the diagnosis of central nervous system infections by underlining their limits to establish an etiological diagnosis.

## Materials and methods

Patients

This was a descriptive, observational, retrospective analysis using data from the Microbiology Laboratory of Mohammed VI University Hospital of Marrakech. This study covered a period of 34 months between March 2018 and December 2020 and included all patients (adults and children) hospitalized in different intensive care units of Mohammed VI University Hospital of Marrakech who underwent a multiplex FilmArray® meningitis/encephalitis panel (FA MEP) PCR on a cerebrospinal fluid (CSF) sample. The Mohammed VI University Hospital of Marrakech is the only tertiary hospital in the region that receives patients referred from the south of Morocco.

This study included all pediatric and adult patients with suspected severe acute meningeal infection for whom the clinician had ordered meningeal PCR for an infectious etiology. Cerebrospinal fluid was collected by lumbar or transfontanellar puncture [1,2].

Methods

After receiving the samples, the culture was performed first with the cytobacteriological examination, followed by a multiplex PCR search for microbial agents. The culture was conducted for all samples, while the PCR was only performed based on microbiological criteria (high cytology, hypoglycorrhachia, and high CSF proteins) or at the request of the clinician.

The cytobacteriological and biochemical study of the CSF was performed according to the standard procedures of the French Society of Microbiology. Protein and glucose concentrations were measured by immunoturbidimetric assay using the Roche/Hitachi Cobas 311 system (Basel, Switzerland) with calibrators and internal controls supplied by Roche and according to the manufacturer’s recommendations.

*FilmArray® Meningitis/Encephalitis Panel (FA MEP) *​​​​*Test*

Approximately 200 μL of CSF was used for the BioFire® FilmArray® meningitis/encephalitis panel PCR panel according to the manufacturer’s recommendations. This multiplex real-time PCR assay consists of an automated nucleic acid assay based on acid extraction, reverse transcription, and nucleic acid amplification, with an average result turnaround time of 75 minutes per run and a handling time of less than five minutes. Transportation of samples from clinical services to the laboratory typically took about 15 minutes.

This PCR detects 14 pathogens: *Cytomegalovirus*,* Enterovirus*,* Herpes simplex virus (HSV) 1/2*,* HHV-6*,* Parechovirus*,* Varicella-zoster virus*,* Cryptococcus neoformans/gattii*,* Escherichia coli *K1,* Haemophilus influenzae*,* Listeria monocytogenes*,* Neisseria meningitidis*,* Streptococcus agalactiae*, and* Streptococcus pneumoniae*.

Statistical analysis

All statistical analyses were performed using the Statistical Package for the Social Sciences (SPSS) version 23.0 (IBM SPSS Statistics, Armonk, NY, USA) and Microsoft Excel (Microsoft Corporation, Redmond, WA, USA). Statistical comparisons were performed using the chi-square test. A probability value (p) of less than 0.05 was considered statistically significant.

## Results

Patient demographics and clinical characteristics

During the research period, CSF samples from 112 patients were analyzed by FilmArray® meningitis/encephalitis panel PCR. The samples were distributed between 2018 and 2020, with an increase in the number of samples tested, ranging from 27 to 44 samples. The average age of the patients was 21.11 years, with age extremes ranging from six days to 73 years. The pediatric population represented more than half of the patients included in the study, with a rate of 54.5%. A slight male predominance was found, with a male/female sex ratio of 1.07. In the ICU, clinical suspicion of meningoencephalitis represented 51.8% (n=58) of the reasons for performing meningeal PCR compared to 43.8% (n=49) for suspicion of meningitis. The overall demographic and clinical characteristics of the patients are summarized in Table 1.

**Table 1:** Demographic and clinical characteristics of patients receiving multiplex PCR in the ICU (n=112) PCR: polymerase chain reaction, ICU: intensive care unit

Features	Number (%)
Years	
2018	27 (24.1)
2019	41 (36.6)
2020	44 (39.3)
Gender	
Male	58 (51.8)
Female	54 (48.2)
Patient age	
<2 months	13 (11.6)
2-23 months	15 (13.4)
2-14 years	33 (29.5)
15-34 years	22 (19.6)
35-64 years	22 (19.6)
>65 years	7 (6.3)
Services	
Neonatal resuscitation	9 (8)
Pediatric resuscitation	50 (44.6)
Vital emergency adult unit	19 (17)
Adult resuscitation	34 (30.4)
Clinical tables	
Meningoencephalitis	58 (51.8)
Meningitis	49 (43.8)
Others	5 (4.4)

FilmArray® meningitis/encephalitis panel PCR

**Figure 1:**
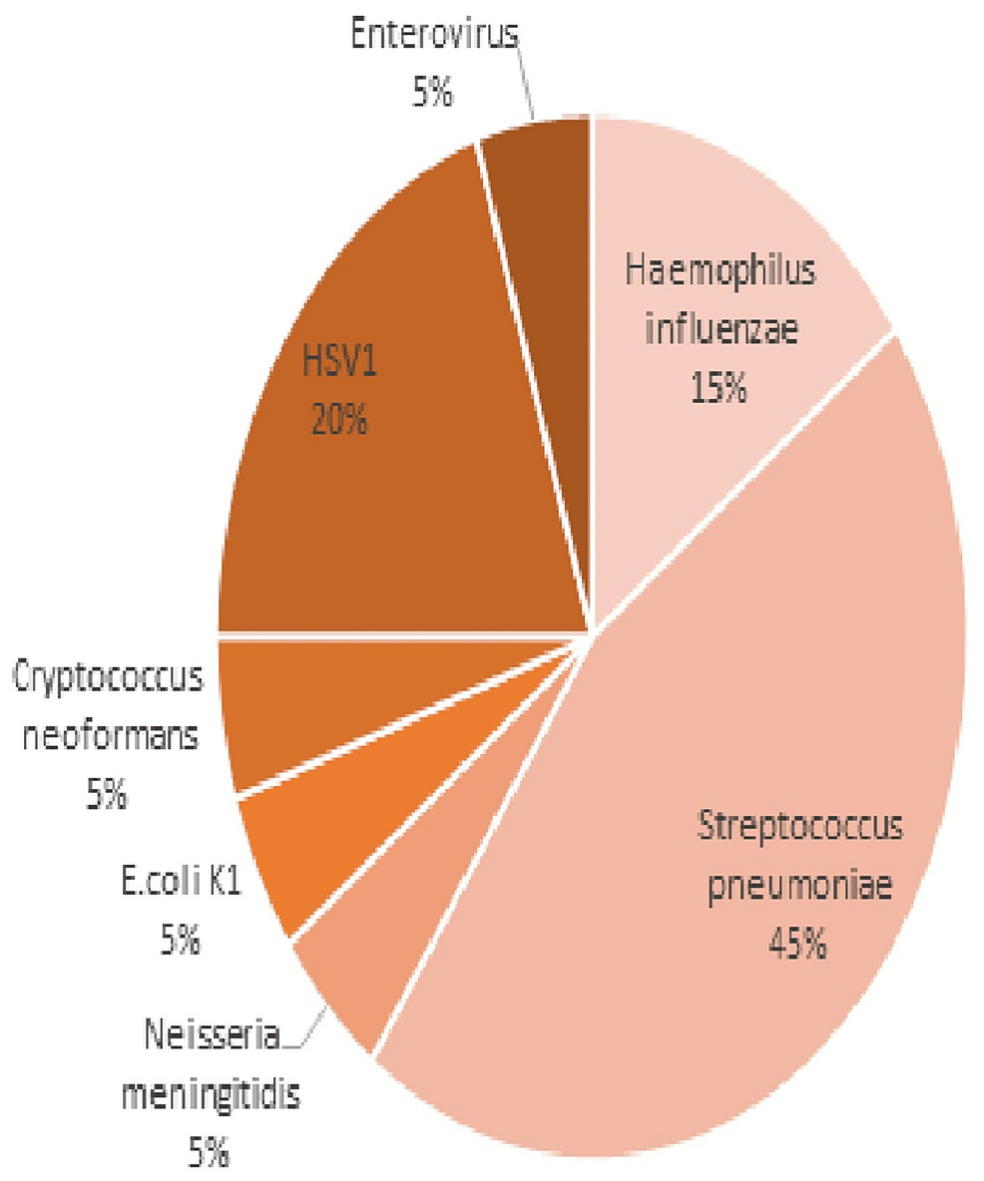
Distribution of pathogens detected by FilmArray® MEP PCR in ICU patients (n=20) MEP: meningitis/encephalitis panel, PCR: polymerase chain reaction, ICU: intensive care unit, *HSV1*: *Herpes simplex virus 1*, *E. coli*: *Escherichia coli*

Among the 112 intensive care unit patients who had a multiplex PCR performed during this time period, 20 (18%) had a positive result, with 14 cases of meningitis and five cases of meningoencephalitis suspected. Bacterial etiology was the most frequent (70%), represented mainly by *Streptococcus pneumoniae* (45%), followed by viruses (25%), with four isolates of *Herpes simplex virus 1*. Only one case of *Cryptococcus neoformans* meningitis was identified in this series (Figure 1). *Streptococcus pneumoniae* and *HSV1 *were commonly found in children and adults. The distribution of agents detected by FilmArray MEP according to patient age categories is shown in Table 2. The time to return the PCR results varied between one hour, 30 minutes, and four hours.

**Table 2:** Distribution of pathogens detected according to age groups of ICU patients (n=20) ICU: intensive care unit, MEP: meningitis/encephalitis panel, PCR: polymerase chain reaction

FilmArray® MEP PCR results								
Pathogens	Number	Percentage (%)	Positives by age group					
			<2 months	2-23 months	2-14 years	15-34 years	35-64 years	>65 years
Bacteria (n=14)								
*Escherichia coli *K1	1	5	0	1	0	0	0	0
Haemophilus influenzae	3	15	1	2	0	0	0	0
Neisseria meningitidis	1	5	0	1	0	0	0	0
Streptococcus pneumoniae	9	45	0	2	3	3	1	0
Viruses (n=5)								
Enterovirus	1	5	1	0	0	0	0	0
Herpes simplex virus 1	4	20	0	0	2	0	1	1
Yeast (n=1)								
Cryptococcus neoformans/gattii	1	5	0	0	0	0	1	0

Cytobacteriological study of CSF

In these intensive care unit patients who underwent multiplex PCR, the cytological study of the CSF revealed that 53.1% of the samples studied had normal cytology (<10 elements/mm^3^). The samples with pleocytosis were subjected to a leukocyte count, which revealed a predominance of polynuclear neutrophils (PNNs) at 59.5% and a predominance of lymphocytes at 32.4% in the studied CSF. Disturbance of the biochemical balance was found in less than 25% of the samples studied. High CSF protein concentrations and hypoglycorrhachia were present in 62.1% and 25.3% of the CSF studied, respectively. The culture was positive in six samples (5.4%) and negative in 94.6%. High levels of CSF protein and hypoglycorrhachia were found in 4/6 of the isolates in culture-positive specimens.

Correlation between FilmArray MEP PCR and culture in the detection of bacteria and yeast

Concordance between the results of the conventional bacterial culture and the FilmArray® meningitis/encephalitis panel PCR was found on 94 samples. Both tests were positive for four samples with the same pathogen isolated in the culture: *Haemophilus influenzae* sensitive to third-generation cephalosporins, *Streptococcus pneumoniae* sensitive to penicillins, multisensitive *Escherichia coli *K1 strain, and *Cryptococcus neoformans*. These four patients each displayed meningitis-related clinical symptoms. However, 90 samples had both negative PCR and culture results. The results of the two techniques disagreed in 18 cases, including 16 suspected neuromeningeal infections recovered only by FA MEP PCR and two diagnoses confirmed only by bacterial cultures of the multiresistant *Acinetobacter baumannii* strain and *Streptococcus oralis *with reduced penicillin sensitivity. The results of the FA MEP PCR and culture concordance according to the clinical picture and the pathogens isolated are shown in Table 3.

**Table 3:** Concordance of culture and PCR by clinical presentation and pathogens in ICU patients PCR-/C-: sample with negative PCR and bacterial culture, PCR+/C-: sample with positive PCR and negative bacterial culture, PCR+/C+: sample with positive PCR and bacterial culture, PCR-/C+: sample with negative PCR and positive bacterial culture, PCR: polymerase chain reaction, ICU: intensive care unit

	PCR-/C-	PCR+/C-	PCR+/C+	PCR-/C+	Total
Clinical suspicion					
Meningoencephalitis	52 (46.4%)	5 (4.5%)	0 (0%)	1 (0.9%)	58 (51.8%)
Meningitis	34 (30.4%)	10 (8.9%)	4 (3.6%)	1 (0.9%)	49 (43.8%)
Other	4 (3.5%)	1 (0.9%)	0 (0%)	0 (0%)	5 (4.4%)
Results					
Negative	90 (80.3%)	0 (0%)	0 (0%)	0 (0%)	90 (80.3%)
Positive					
Haemophilus influenzae	0 (0%)	2 (1.8%)	1 (0.9%)	0 (0%)	3 (2.7%)
Streptococcus pneumoniae	0 (0%)	8 (7.1%)	1 (0.9%)	0 (0%)	9 (8%)
Herpes simplex virus 1	0 (0%)	4 (3.6%)	0 (0%)	0 (0%)	4 (3.6%)
Enterovirus	0 (0%)	1 (0.9%)	0 (0%)	0 (0%)	1 (0.9%)
Cryptococcus neoformans	0 (0%)	0 (0%)	1 (0.9%)	0 (0%)	1 (0.9%)
Neisseria meningitidis	0 (0%)	1 (0.9%)	0 (0%)	0 (0%)	1 (0.9%)
*Escherichia coli *K1	0 (0%)	0 (0%)	1 (0.9%)	0 (0%)	1 (0.9%)
*Acinetobacter* spp.	0 (0%)	0 (0%)	0 (0%)	1 (0.9%)	1 (0.9%)
Streptococcus oralis	0 (0%)	0 (0%)	0 (0%)	1 (0.9%)	1 (0.9%)
Total	90 (80%)	16 (14.3%)	4 (3.6%)	2 (2%)	112 (100%)

The cytological study of the CSF allowed the conclusion of the presence or absence of pleocytosis for each of the samples. The absence of pleocytosis was found to be a reliable indicator of the negativity of both the PCR and the bacterial culture (p<0.0001). Table 4 groups the distribution of PCR and culture data according to pleocytosis’ presence or absence. CSF hypercellularity was related to abnormalities in CSF biochemical markers such as high protein levels in all but one of the positive PCRs, although hypoglycorrhachia was identified in only 53.3% of the PCR-positive samples.

**Table 4:** Prevalence of pleocytosis according to PCR and culture results in ICU patients PCR-/C-: sample with negative PCR and bacterial culture, PCR+/C-: sample with positive PCR and negative bacterial culture, PCR+/C+: sample with positive PCR and bacterial culture, PCR-/C+: sample with negative PCR and positive bacterial culture, PCR: polymerase chain reaction, ICU: intensive care unit p<0.0001

	PCR-/C-	PCR+/C-	PCR+/C+	PCR-/C+	Total
White blood cell count					
<10 cells/mm^3^	64 (57.1%)	1 (0.9%)	0 (0%)	1 (0.9%)	66 (58.9%)
>10 cells/mm^3^	26 (23.2%)	15 (13.4%)	4 (3.6%)	1 (0.9%)	46 (41.1%)
Total	90 (80.3%)	16 (14.3%)	4 (3.6%)	2 (1.8%)	112 (100%)

All cases of viral meningitis or meningoencephalitis were identified solely by PCR. Of the confirmed viral meningitis by PCR, three cases out of five had clinical, cytological, and biochemical concordance. In a 36-year-old HIV+ immunocompromised patient, *Cryptococcus neoformans *was recovered as the causative agent of meningitis from the only CSF sample with a positive PCR and culture with normal cytology and biochemistry. The two most often isolated agents (28.5%) that caused meningitis and/or meningoencephalitis with lymphocytic formula were *HSV-1 *and *Streptococcus pneumoniae*.

The etiology varied depending on the clinical suspicion; for suspected meningitis, bacteria were more involved than viruses and conversely for suspected meningoencephalitis. The pathogen distribution according to the clinic is outlined in Table 5. No coinfections were detected using the two methods.

**Table 5:** PCR results according to clinical presentation in ICU patients PCR: polymerase chain reaction, ICU: intensive care unit

	Meningitis	Meningoencephalitis	Other	Total
Negative	34 (30.4%)	52 (46.4%)	4 (3.5%)	90 (80.3%)
Positive	15 (13.4%)	6 (5.4%)	1 (0.9%)	22 (19.7%)
*Haemophilus* *influenzae*	3 (2.7%)	0 (0%)	0 (0%)	3 (2.7%)
Streptococcus pneumoniae	8 (7.1%)	1 (0.9%)	0 (0%)	9 (8%)
*Escherichia* *coli* K1	1 (0.9%)	0 (0%)	0 (0%)	1 (0.9%)
Neisseria meningitidis	0 (0%)	0 (0%)	1 (0.9%)	1 (0.9%)
Acinetobacter baumannii	1 (0.9%)	0 (0%)	0 (0%)	1 (0.9%)
Streptococcus oralis	0 (0%)	1 (0.9%)	0 (0%)	1 (0.9%)
Herpes simplex virus 1	0 (0%)	4 (3.6%)	0 (0%)	4 (3.6%)
Enterovirus	1 (0.9%)	0 (0%)	0 (0%)	1 (0.9%)
Cryptococcus neoformans	1 (0.9%)	0 (0%)	0 (0%)	1 (0.9%)
Total	49 (43.8%)	58 (51.8%)	5 (4.4%)	112 (100%)

## Discussion

Meningitis and/or meningoencephalitis can have similar clinical signs, making it difficult to differentiate between bacterial, viral, or fungal etiologies, which require specific and specialized management. The admission of patients with suspected neuromeningeal infections to the intensive care unit can reach half and will depend on the etiology, severity of the illness, and speed of management [10,11]. As a result, a delayed diagnosis might have serious clinical consequences for both the patient and those around him: unnecessary first-line broad-spectrum antibiotic treatment for certain etiologies, postponing adequate antimicrobial treatment, the risk of developing antibiotic resistance, and a delay in initiating prophylactic treatment for contact subjects [12]. Thus, the etiological diagnosis remains a real emergency for both the clinician and the biologist. Conventional bacterial and fungal culture techniques can stall this management, with a time limit of at least 48 hours before results are available. These conventional diagnostic methods are technically complex, lack sensitivity and specificity, and also require scientific expertise [13]. Their accuracy may be affected by the administration of antibiotics. This risk is overcome by molecular biology PCR techniques, which allow rapid diagnosis, in particular the FilmArray® meningitis/encephalitis panel test, whose contribution was evaluated in this study, in the etiological diagnosis of neuromeningeal infections in the intensive care unit [14].

This retrospective study included 112 patients hospitalized in different intensive care units, whose CSF samples were simultaneously tested for the presence of 14 pathogens detected by FilmArray® MEP and then correlated with bacterial and fungal cultures. The intensive care units’ demand for FA MEP has grown since it was introduced in March 2018 at the microbiology department of Mohammed VI Hospital, going from 27 samples examined in 2018 to 44 in 2020. Regarding the demographic profile of the patients, a slight male predominance was observed, which is in line with the results obtained in the United States [12], China [13], and Morocco [15] and in contrast to the results from Brazil [16] and Ireland [17], where women were more predominant. As opposed to other research, the pediatric population was the most represented with a rate of 54.5% [12,15,18]. Meningoencephalitis was the most prevalent reason for requesting multiplex PCR, at 51.8%.

In the present research, the overall positivity rate observed with FilmArray® MEP in the ICU was 18%, similar to those described by other authors [14,19]. This low positive rate might be explained by the presence of various etiologies, particularly inflammatory ones, which can cause meningitis and/or meningoencephalitis in patients treated in the ICU. In a total of 20 cases of meningitis documented microbiologically by PCR, bacteria represented 70% of all pathogens found, contrary to published studies where viruses were prevailing [14,16,19]. The most isolated bacterium was *Streptococcus pneumoniae* (nine isolates), followed by *Haemophilus influenzae*, which is consistent with the results of the study by Tarai et al. [14]. *Enterovirus* was the representative virus in several studies, while it was identified in this series only once. The FA MEP test was the only technique that diagnosed* Cryptococcus neoformans/gattii* meningitis in a patient with HIV immunosuppression. Normal cytology and biochemistry contrasting with clinical expression and positive PCR and culture is a frequent situation in such a context and has no diagnostic value [20]. The sensitivity of FA MEP has been demonstrated to be 100% for the detection of *Cryptococcus* at >100 CFU/mL [12]. This study has found low detection rates of *Enterovirus* and *Cryptococcus neoformans/gattii* in intensive care unit patients’ CSF. *Enterovirus* meningitis is often identified in children and is typically associated with mild symptoms that do not need extensive care. These patients are often admitted to the pediatric unit. Meningitis caused by *Cryptococcus neoformans* is often diagnosed in immunocompromised patients and treated by our hospital’s infectious diseases department. Symptoms may not usually need critical care unit admission.

Bacterial and fungal cultures were positive in six patients (5.6%), with a concordant PCR result in only four cases. This positivity rate is lower than that reported in previous studies using only culture, such as the studies of Ramesh et al. [21], where 25% of samples were culture-positive. The culture was able to isolate bacteria and yeast from four of the 15 patients who had a positive multiplex PCR, representing 26.6% of all PCR-confirmed patients. This matches the results of Du et al. who concluded that the FilmArray® MEP had a relatively higher sensitivity than conventional diagnostic methods, including culture, especially after the administration of antimicrobial therapy [13]. In the ICU, during this period, two cases of meningitis were confirmed only by culture, while PCR was negative. These results are explained by the limitations of FA MEP detection in comparison to bacterial and fungal culture, particularly when microbial agents not included in the panel are involved, as was the case in these two patients with the isolation of *Acinetobacter baumannii* and *Streptococcus oralis* in culture. Since nosocomial pathogens of meningitis are not included in the FilmArray® MEP, a negative PCR result cannot rule out the diagnosis of nosocomial bacterial meningitis. These false negatives are particularly limiting for nosocomial bacteria. The biologist needs to be careful while interpreting such a false-negative result. In light of this, clinical information must accompany each request for a paraclinical evaluation pointing to a community or nosocomial context.

Regarding the cytological research, CSF pleocytosis was present in all recovered infections, with the exception of one *HSV1* isolate. This finding demonstrates a statistically significant relationship (p<0.001), given that the absence of pleocytosis predicted a negative multiplex PCR result in 65.4% of cases. This conclusion was partially confirmed by Naccache et al., who stated that pleocytosis was predictive of the ability to confirm the presence of bacterial agents and not viruses [19].

The agreement of FA MEP PCR and culture results was observed only for clinical suspicions of meningitis, whereas meningoencephalitis was mostly confirmed by PCR with *HSV1* as the major pathogen. Consequently, it is crucial to note that each PCR and culture result must be interpreted based on a clinical and biological set of arguments. As is the case in certain circumstances, some viruses may be found in a latent condition for which treatment is not warranted, posing a risk of medication toxicity [22].

To summarize, the FilmArray MEP confirmed 20 cases of probable neuromeningeal infection, accounting for 17.9% of all samples collected, with 16 (14.3%) recovered only by PCR. This comparative study of FA MEP and culture in ICU patients highlighted the critical role of this diagnostic tool using the syndromic approach. It was able to quickly confirm the diagnosis in 14.3% of the patients whose CSF culture was sterile, rapidly distinguish between viral, bacterial, and fungal etiologies, and instantly orient the therapeutic management in ICU, thus concluding that there was no neuromeningeal infection based on a negative culture would have been dangerous, particularly in patients with an unstable and critical clinical state that may have deteriorated in the absence of a specific diagnosis and adequate management. This study also revealed important FA MEP use limits that every doctor and biologist should know about: false negatives due to non-detection of pathogens not included in the panel; false positives are related to the presence of certain viruses in trace amounts, particularly for human herpesvirus 6 (HHV6) and CMV [18]; and the inability to establish whether the detected pathogens are sensitive to antibiotics and determine the degree of their resistance.

Consequently, culture is still necessary for all CSF samples, regardless of PCR results.

The main limitation of this study was the lack of comprehensive clinical data to assess the overall impact of the use of multiplex PCR, including the cost-effectiveness and length of stay in the ICU and hospital.

## Conclusions

This study highlighted the important impact of multiplex PCR on the early diagnostic and targeted therapeutic management of infectious meningitis and meningoencephalitis in intensive care unit patients. In similar conditions, this syndromic rapid diagnostic test should be used as a first-line tool for suspected central nervous system infection. It allows a quick and specific diagnosis by screening for 14 pathogens in a single test, with the prospect of receiving rapid results within three hours, from the transport of the sample to the clinician’s information. Nonetheless, its contribution in extreme emergency situations may be justifiable despite its prohibitive price, especially for developing countries. PCR and culture of CSF with conventional methods represent two complementary tools that must be performed synergistically to increase the sensitivity of the results, thus allowing better management of central nervous system infections in patients in critical care units. Further studies are needed to define its impact on therapeutic decision-making and the cost-effectiveness of its routine use in managing central nervous system infections in the ICU.

## References

[1] Edmond K, Clark A, Korczak VS, Sanderson C, Griffiths UK, Rudan I (2010). Global and regional risk of disabling sequelae from bacterial meningitis: a systematic review and meta-analysis. Lancet Infect Dis.

[2] Brouwer MC, Tunkel AR, van de Beek D (2010). Epidemiology, diagnosis, and antimicrobial treatment of acute bacterial meningitis. Clin Microbiol Rev.

[3] Boucher A, Herrmann JL, Morand P, Buzelé R, Crabol Y, Stahl JP, Mailles A (2017). Epidemiology of infectious encephalitis causes in 2016. Med Mal Infect.

[4] Wright C, Wordsworth R, Glennie L (2013). Counting the cost of meningococcal disease: scenarios of severe meningitis and septicemia. Paediatr Drugs.

[5] Stahl JP, Azouvi P, Bruneel F (2017). Guidelines on the management of infectious encephalitis in adults. Med Mal Infect.

[6] Hoen B, Varon E, Debroucker T, Fantin B, Grimprel E, Wolff M, Duval X (2019). Management of acute community-acquired bacterial meningitis (excluding newborns). Short text. Med Mal Infect.

[7] Águeda S, Campos T, Maia A (2013). Prediction of bacterial meningitis based on cerebrospinal fluid pleocytosis in children. Braz J Infect Dis.

[8] Visseaux B, Armand-Lefèvre L (2019). Syndromic multiplex approach in intensive care (Article in French). Méd Intensive Réa.

[9] Mailles A, Stahl JP (2009). Infectious encephalitis in france in 2007: a national prospective study. Clin Infect Dis.

[10] John R, Hirsch N (2015). Central nervous system infections in intensive care patients. Anaesth Intensive Care Med.

[11] Zeighami H, Roudashti S, Bahari S, Haghi F, Hesami N (2021). Frequency of etiological agents of acute bacterial meningitis using culture and polymerase chain reaction assay. New Microbes New Infect.

[12] Leber AL, Everhart K, Balada-Llasat JM (2016). Multicenter evaluation of BioFire FilmArray meningitis/encephalitis panel for detection of bacteria, viruses, and yeast in cerebrospinal fluid specimens. J Clin Microbiol.

[13] Du B, Hua C, Xia Y (2019). Evaluation of the BioFire FilmArray meningitis/encephalitis panel for the detection of bacteria and yeast in Chinese children. Ann Transl Med.

[14] Tarai B, Das P (2019). FilmArray® meningitis/encephalitis (ME) panel, a rapid molecular platform for diagnosis of CNS infections in a tertiary care hospital in North India: one-and-half-year review. Neurol Sci.

[15] Adil R, Taoufik R, Fatima I (2019). Contribution of the syndromic approach in the diagnosis of meningitis at the university hospital of Marrakech. PAMJ Clin Med.

[16] Domingues RB, Santos MV, Leite FB, Senne C (2019). FilmArray Meningitis/Encephalitis (ME) panel in the diagnosis of bacterial meningitis. Braz J Infect Dis.

[17] Mostyn A, Lenihan M, O'Sullivan D (2020). Assessment of the FilmArray® multiplex PCR system and associated meningitis/encephalitis panel in the diagnostic service of a tertiary hospital. Infect Prev Pract.

[18] Vincent JJ, Zandotti C, Baron S (2020). Point-of-care multiplexed diagnosis of meningitis using the FilmArray® ME panel technology. Eur J Clin Microbiol Infect Dis.

[19] Naccache SN, Lustestica M, Fahit M, Mestas J, Dien Bard J (2018). One year in the life of a rapid syndromic panel for meningitis/encephalitis: a pediatric tertiary care facility’s experience. J Clin Microbiol.

[20] Kapila K, Sharma YV, Kotwal J, Banerjee A, Kaur J (2003). Cryptococcal meningitis: a clinicopathological account of seven cases encountered in a military setting. Med J Armed Forces India.

[21] Ramesh ST, Girish Babu RJ (2016). Pathological and microbiological analysis of cerebrospinal fluid in bacterial meningitis. Trop J Pathol Microbiol.

[22] Gomez CA, Pinsky BA, Liu A, Banaei N (2017). Delayed diagnosis of tuberculous meningitis misdiagnosed as herpes simplex virus-1 encephalitis with the FilmArray syndromic polymerase chain reaction panel. Open Forum Infect Dis.

